# Evaluation of hospital outcomes: the relation between length-of-stay, readmission, and mortality in a large international administrative database

**DOI:** 10.1186/s12913-018-2916-1

**Published:** 2018-02-14

**Authors:** Hester F. Lingsma, Alex Bottle, Steve Middleton, Job Kievit, Ewout W. Steyerberg, Perla J. Marang-van de Mheen

**Affiliations:** 1 Department of Public Health, Erasmus Medical Centre, PO box 2040, 3000 CA Rotterdam, The Netherlands; 2 Imperial College, Faculty of Medicine, School of Public Health, South Kensington Campus, London, SW7 2AZ UK; 3 Dr Foster Intelligence, 3 Dorset Rise, London, EC4Y 8EN UK; 4 Department of Medical Decision Making, Leiden University Medical Centre, Albinusdreef 2, 2333 ZA Leiden, The Netherlands

**Keywords:** Benchmarking, Quality of care, Outcomes, Ordinal models, Composite outcomes, Administrative data

## Abstract

**Background:**

Hospital mortality, readmission and length of stay (LOS) are commonly used measures for quality of care. We aimed to disentangle the correlations between these interrelated measures and propose a new way of combining them to evaluate the quality of hospital care.

**Methods:**

We analyzed administrative data from the Global Comparators Project from 26 hospitals on patients discharged between 2007 and 2012. We correlated standardized and risk-adjusted hospital outcomes on mortality, readmission and long LOS. We constructed a composite measure with 5 levels, based on literature review and expert advice, from survival without readmission and normal LOS (best) to mortality (worst outcome). This composite measure was analyzed using ordinal regression, to obtain a standardized outcome measure to compare hospitals.

**Results:**

Overall, we observed a 3.1% mortality rate, 7.8% readmission rate (in survivors) and 20.8% long LOS rate among 4,327,105 admissions. Mortality and LOS were correlated at the patient and the hospital level. A patient in the upper quartile LOS had higher odds of mortality (odds ratio = 1.45, 95% confidence interval 1.43–1.47) than those in the lowest quartile. Hospitals with a high standardized mortality had higher proportions of long LOS (r = 0.79, *p* < 0.01). Readmission rates did not correlate with either mortality or long LOS rates. The interquartile range of the standardized ordinal composite outcome was 74–117. The composite outcome had similar or better reliability in ranking hospitals than individual outcomes.

**Conclusions:**

Correlations between different outcome measures are complex and differ between hospital- and patient-level. The proposed composite measure combines three outcomes in an ordinal fashion for a more comprehensive and reliable view of hospital performance than its component indicators.

## Background

Outcome measures for quality of care are increasingly used to monitor and compare hospital performance, with the aim to identify areas for improvement. Three outcome measures that are commonly used in various countries to evaluate quality of care in hospitals are in-hospital mortality, readmissions, and long length of stay (LOS) [1]. However, these outcomes are interrelated which will affect the interpretation of hospital outcomes. For example, patients who die in hospital cannot be readmitted, so that in theory hospitals may have low readmission rates due to relatively high mortality. Such mechanisms need to be understood for outcome measures to be able to reflect quality of care.

A previous study among Medicare patients hospitalized for acute myocardial infarction, heart failure or pneumonia did not find an association for mortality and readmission, except a weak association for heart failure patients [2]. However, this study only examined hospital-level and not patient-level associations. Both types of associations need to be considered to evaluate whether interventions to reduce mortality may raise readmission rates or whether the two measures have shared underlying processes.

Furthermore, to obtain a comprehensive picture of quality –especially for between-hospital comparisons - it is attractive to jointly report outcome measures as e.g. mortality may be very low in some patient groups but readmissions are common. In addition, combining multiple outcome measures has the advantage of an increased number of events per hospital, resulting in more reliable estimates (lower statistical uncertainty) and thereby more reliable comparisons of hospitals [3].

In this study we aim to 1) disentangle the relationship between mortality, readmission and long LOS both at patient and hospital level, and 2) to develop a measure to jointly report the three outcomes for a more comprehensive, unambiguous and more reliable estimate of hospital-specific quality of care to be used within a hospital over time and between-hospital comparisons.

## Methods

### Data

We used data from the Global Comparators Project in which hospitals from various countries collaborate and share their routinely collected administrative admission data. Participating hospitals were large academic medical units, likely to be fairly comparable with respect to their (complex) patient population. Diagnoses were combined into Clinical Classification Software (CCS) groups, procedures into groups representing major surgical specialties and comorbidities defined for the various coding systems as described elsewhere [1]. For the present study, data from 26 hospitals in six countries (USA, Netherlands, UK, Italy, Belgium and Australia) were included who agreed their data to be used for research.

Within the Global Comparators Project, clinicians and other professionals work in Global Outcomes Accelerated Learning (GOAL) groups to use the observed variation in outcomes to initiate inquiries to identify best practices that participants may implement at their own institutions. Therefore, in addition to studying all patients, we focused on 3 specific GOAL areas: patients with stroke, heart failure or colorectal surgery as their principal diagnosis or procedure during admission. We expected these clinical areas to differ in having relatively high mortality rates (e.g. stroke), or that readmission would be a more relevant quality indicator (e.g. heart failure). All patients discharged in the years 2007 to 2012 were included in the analysis.

### Definition of variables

We considered three outcomes: mortality, readmission, and long LOS. Mortality was defined as death in hospital during the index admission. Readmission was defined as an unplanned (emergency) readmission to the same hospital within 30 days after discharge. Long LOS was defined as a LOS greater than the 75th percentile for the specific diagnosis or procedure group (upper-quartile LOS). In a sensitivity analysis, long LOS was defined as greater than the 90th percentile LOS (upper-decile LOS) to assess whether this affected the results.

### Statistical analysis

For case-mix adjustment, separate logistic regression models were developed for each of the 259 diagnosis and 32 procedure groups for each of the three outcome measures, using the inbuilt backward elimination procedure in SAS with all case-mix variables included and retaining variables with *p* < 0.1. Case-mix variables were used as described previously [1]: age-group, sex, method of admission (planned / unplanned), transferred in from other hospital, urgent admission in previous month, Elixhauser comorbidity score, year, diagnosis (sub)group or procedure group (for analysis of all patients). Statistical interactions between age and Elixhauser comorbidity score, and between method of admission and transfer, were included as candidates, based on a priori beliefs [1]. To aid model convergence, age groups with fewer than 10 events were iteratively combined with the immediately older group [4]. This resulted in expected probabilities per outcome for each patient. At a hospital level, these probabilities were summed to obtain the expected number for a particular outcome. By dividing the observed number by the expected number and multiplying by 100, standardized ratios of mortality, readmission, and long LOS per hospital were calculated.

At a patient level, we first assessed the correlation between outcomes using logistic regression models with mortality and readmission as dependent variables and upper quartile LOS as independent variable, both unadjusted and adjusted for center (as fixed effect) and case-mix. The resulting odds ratios indicate the increase in odds of mortality or readmission for patients in the upper quartile LOS. At a hospital level, we assessed the correlation between standardized mortality, readmission (survivors) and long LOS (survivors) using Pearson correlation coefficients.

We then created a composite outcome measure in which the different combinations of outcomes were ordered from best to worst outcome: 1) alive, no long LOS, no readmission, 2) alive, long LOS, no readmission, 3) alive, no long LOS, readmission, 4) alive, long LOS, readmission, 5) death. This ordering was based on previous research showing that adverse outcomes occurring during admission, thereby prolonging LOS, did not affect patients’ evaluation of quality of care [5]. Adverse outcomes that occurred after discharge, often resulting in readmission, negatively affected patients’ evaluation of quality of care. This motivates our ordering with a readmission being worse than long LOS. The ordering was presented at a meeting of about 100 experts, clinicians and CEOs involved in the Global Comparators Project, for agreement. The composite outcome measure with 5 levels was analyzed with ordinal logistic regression with the case-mix variables and hospital as a fixed effect to estimate a coefficient per hospital, representing the standardized effect of hospitals on the composite outcome. This coefficient was then used to calculate the standardized rate, by calculating the average of all hospitals coefficients and then calculating the difference of each hospital coefficient compared with that average. Exponentiating this difference will give an odds ratio which can be interpreted as higher or lower than the average. We assessed the correlation between the standardized rates of the individual versus the composite outcome.

Finally, we assessed whether this composite measure would enable us to better discriminate between hospitals in terms of their apparent performance. We thereby evaluated the reliability of ranking the hospitals (the rankability) [3]. This measure has been proposed to quantify the signal (i.e. differences in outcome) versus noise in hospital rankings. Rankability consists of two elements: 1) The magnitude of the between-hospital differences, defined by tau [2], the variance of the random hospital effects, and 2) the uncertainty in the individual hospital estimates, defined as the median sigma, the squared standard error of the fixed effect hospital estimates. The rankability is a percentage and can be interpreted as the part of the total between-hospital differences that is due to ‘true’ differences in outcome (rather than being due to statistical uncertainty). It is thus a characteristics of the group of hospitals we aim to compare, not of each single hospital. A logistic regression model was fitted with hospital included as a random effect to estimate tau [2]. To estimate the second element, the same regression model was fitted with hospital included as a fixed effect. This was done both for the individual and the composite outcome.

## Results

### Patient population and outcome

In total 4,327,105 patients were included. Most patients were from the USA (42%) and the UK (24%, Table 1).

**Table 1:** Baseline patient characteristics and outcome (N(%) or Mean (SD))

	All patients *N* = 4,327,105	Stroke patients *N* = 83,163	Colorectal patients *N* = 35,537	Heart Failure patients *N* = 85,024
Country				
Australia	183,009 (4.2%)	3373 (4.1%)	1374 (3.9%)	3348 (3.9%)
Belgium	303,620 (7.0%)	3217 (3.9%)	3246 (9.1%)	4018 (4.7%)
UK	1,227,454 (28.4%)	20,207 (24.3%)	7437 (20.9%)	15,730 (18.5%)
Italy	174,970 (4.0%)	5340 (6.4%)	1527 (4.3%)	6811 (8.0%)
Netherlands	601,841 (13.9%)	12,963 (15.6%)	4078 (11.5%)	5774 (6.8%)
USA	1,836,211 (42.4%)	38,063 (45.8%)	17,875 (50.3%)	49,343 (58.0%)
Year of discharge				
2007	798,924 (18.5%)	14,493 (17.4%)	6510 (18.3%)	15,249 (17.9%)
2008	847,656 (19.6%)	15,911 (19.1%)	6743 (19.0%)	16,286 (19.2%)
2009	862,412 (19.9%)	16,320 (19.6%)	6826 (19.2%)	16,196 (19.1%)
2010	864,918 (20.0%)	16,861 (20.3%)	6973 (19.6%)	16,303 (19.2%)
2011	678,676 (15.7%)	13,620 (16.4%)	5939 (16.7%)	14,286 (16.8%)
2012	274,519 (6.3%)	5958 (7.2%)	2546 (7.2%)	6704 (7.9%)
Age	49.0 (26.2)	68.6 (15.8)	58.8 (18.8)	70.0 (16.7)
Male gender	1,985,545 (45.9%)	42,861 (51.5%)	17,531 (49.3%)	45,296 (53.3%)
Number of comorbidities	1.4 (1.7)	2.2 (1.9)	1.6 (1.6)	3.8 (2.2)
Unplanned	2,740,694 (63.3%)	69,015 (83.0%)	10,932 (30.8%)	72,460 (85.2%)
Urgent	2,269,541 (52.5%)	66,990 (80.6%)	10,638 (29.9%)	72,086 (84.8%)
Outcomes				
Mortality	131,791 (3.1%)	11,308 (13.6%)	1762 (5.0%)	5681 (6.7%)
Readmission (overall)	325,663 (7.5%)	5264 (6.3%)	3576 (10.1%)	13,428 (15.8%)
Readmission (survivors)*	325,663 (7.8%)	5264 (7.3%)	3576 (10.6%)	13,428 (16.9%)
LOS (days)	6.7 (11.7)	12.0 (19.1)	14.2 (18.3)	8.8 (11.5)
Long LOS (upper quartile)	901,657 (20.8%)	17,177 (20.7%)	8046 (22.6%)	17,591 (20.7%)

*% calculated over survivors: 4,195,314 (all patients) 71,855 (stroke patients) 33,775 (colorectal patients) 79,343 (heart failure patients)

The overall crude mortality rate was 3.1%, and among the patients discharged alive 7.8% were readmitted. The mean LOS over the period 2007–2012 was 6.7 days. The three selected patient groups differed considerably both in case-mix of patients and in outcomes. Heart failure patients were the oldest (mean age 70) and had most comorbidities (mean 3.8 comorbidities). Their in-hospital mortality rate was 6.7%, LOS was shorter than in the other patient groups (mean 8.8 days) and readmissions occurred frequently (16.9%). Mortality was higher among stroke patients (13.6%) and colorectal patients had the longest LOS (mean 14.2 days).

Patients who were in the upper quartile LOS had an increased odds of mortality, particularly among heart failure patients (Table 2). The exception was in stroke patients, where a long LOS was associated with lower odds of mortality. We found that mortality occurred usually early after stroke so that patients who die have a short LOS. Patients with longer LOS had a significantly higher odds of readmission in all patient groups.

**Table 2:** Unadjusted and adjusted effects of LOS on mortality and readmission (patient level), unadjusted and with adjustment for centre and case-mix

	Mortality Unadjusted OR (95% CI)	Adjusted OR (95% CI)	Readmission Unadjusted OR (95% CI)	Adjusted OR (95% CI)
All patients				
Long LOS (quartile)	1.96 (1.93–1.98)	1.45 (1.43–1.47)	1.53 (1.52–1.55)	1.37 (1.35–1.38)
Stroke patients				
Long LOS (quartile)	0.68 (0.64–0.72)	0.46 (0.43–0.49)	1.33 (1.25–1.42)	1.16 (1.08–1.25)
Colorectal patients				
Long LOS (quartile)	1.77 (1.60–1.96)	1.31 (1.16–1.47)	1.41 (1.31–1.53)	1.34 (1.23–1.45)
Heart Failure patients				
Long LOS (quartile)	2.33 (2.20–2.46)	1.38 (1.29–1.47)	1.17 (1.12–1.23)	1.17 (1.11–1.23)

OR = odds ratio, CI = confidence interval

### Variation between hospitals

At a hospital level, there was a large variation between hospitals both in case-mix and in outcomes (Table 3). The interquartile range (IQR) for urgent admissions was 35%–64% and 0.59–1.92 for the average number of comorbidities. There was also substantial variation in outcome, both in observed outcomes and in expected outcomes as a result of the case-mix differences. As a result the standardized outcomes also varied considerably. The smallest variation was shown in standardized readmission rates, and the largest variation for standardized mortality rates, specifically for colorectal (IQR 68–169) and heart failure patients (IQR 48–193).

**Table 3:** Baseline characteristics and outcome per hospital (n = 26), median and Inter Quartile Range (IQR)

	All patients	Stroke patients	Colorectal patients	Heart Failure patients
Number of admissions	152,429 [109,342–225,135]	3295 [1905–4113]	1146 [709–2005]	3148 [825–5037]
Age	48.5 [45.7–50.8]	67.5 [64.6–71.9]	57.7 [55.5–61.6]	70.1 [64.6–75.4]
Male gender	46.1% [44.4%–50.1%]	51.7% [49.3%–54.5%]	48.1% [46.9%–53.2%]	54.2% [50.6%–56.6%]
Number of comorbidities	1.01 [0.59–1.92]	1.93 [1.08–3.02]	1.23 [0.87–1.82]	2.54 [2.17–4.74]
Unplanned	65.0% [37.6%–74.4%]	81.7% [76.0%–90.2%]	30.9% [21.7%–37.6%]	85.7% [67.6%–94.1%]
Urgent	49.0% [34.9%–63.5%]	80.2% [74.1%–85.6%]	29.0% [21.7%–35.7%]	85.2% [67.7%–93.6%]
Outcomes				
Mortality	2.6% [2.2%–3.3%]	14.1% [11.7%–16.4%]	5.6% [3.3%–7.1%]	7.9% [2.9%–12.9%]
Expected mortality	3.1% [2.0%–3.5%]	13.5% [12.6%–15.2%]	4.4% [3.4%–5.3%]	6.3% [5.1%–8.7%]
Standardized mortality	122 [72–132]	105 [88–117]	118 [68–169]	125 [48–193]
Readmission (survivors)	7.6% [4.8%–8.9%]	6.0% [4.5%–8.9%]	11.0% [9.0%–11.9%]	15.5% [11.3%–17.7%]
Expected readmission	7.7% [6.0%–9.4%]	7.8% [5.8%–8.2%]	10.4% [10.1%–11.9%]	16.7% [13.7%–19.0%]
Standardized readmission	95 [82–104]	84 [62–105]	97 [85–113]	95 [83–104]
Upper quartile LOS	22.2% [17.0%–25.0%]	19.3% [13.4%–30.6%]	28.0% [15.9%–34.0%]	21.5% [15.3%–32.3%]
Expected upper quartile LOS	21.2% [19.8%–23.1%]	23.6% [20.5%–25.8%]	23.5% [21.8%–24.3%]	22.6% [20.1%–23.9%]
Standard. upper quartile LOS	105 [69–118]	97 [53–121]	130 [70–141]	113 [64–143]

**Fig. 1:**
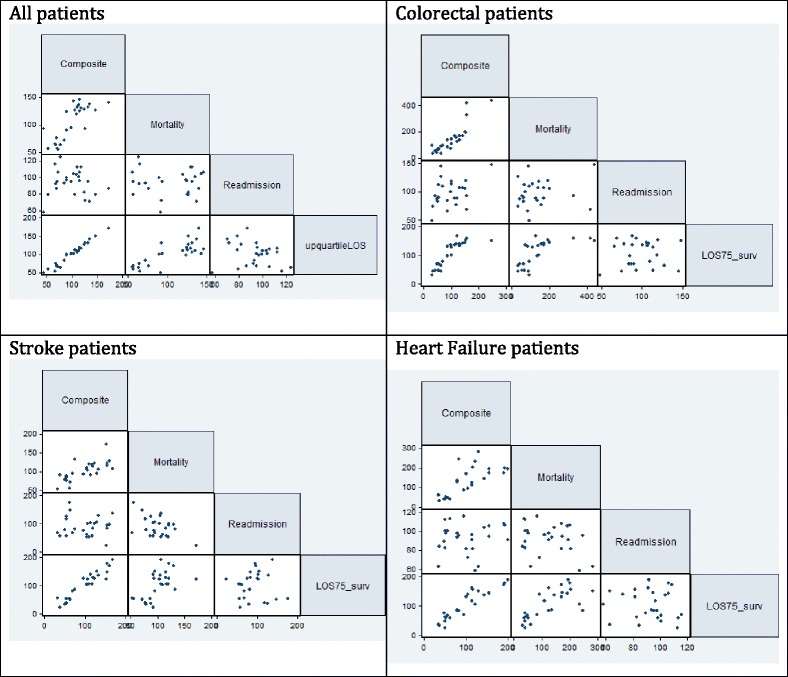
Correlations between standardized rates of composite outcome and individual outcomes at hospital level

For the relationships between standardized outcomes at a hospital level (Fig. 1, Table 4), the strongest (positive) correlation was seen between mortality and long LOS, indicating that hospitals with more long LOS patients also had higher mortality rates (r = 0.79, *p* < 0.01). This agrees with the associations found at a patient level, where patients with longer LOS also had higher mortality probability. However, this positive correlation was found for all diagnosis groups, including stroke where at a patient level the patients with long LOS had lower mortality rates. This may be related to the fact that there is a group of patients that die early during admission, consistent with the associations found on patient level, and a group of patients who survive. So the hospitals that have a relatively large group of stroke patients who die are the same hospitals in which the survivors have a long length of stay, resulting in the positive correlation at a hospital level but involving different groups of patients. An alternative explanation might be that the same patient level associations are found in all hospitals, but that the average LOS differs across hospitals resulting in a reversed hospital level association. In addition, positive correlations were found between long LOS among survivors and long LOS among patients who die (r = 0.77, *p* < 0.01). This indicates that hospitals have a long LOS among both surviving and dying patients, so that it seems to be a characteristic of those hospitals. In contrast, correlations of readmission with mortality (r = − 0.06, *p* = 0.76) and long LOS (r = − 0.20, *p* = 0.30) were both not significant, indicating that readmissions do not cluster in the same hospitals as mortality or long LOS.

**Table 4:** Correlations of composite with mortality, readmission (survivors), upper quartile LOS (survivors)

	Mortality	Readmission	Upper quartile LOS
All patients	r = 0.78 p < 0.01	r = −0.07 *p* = 0.72	r = 0.98 *p* < 0.01
Stroke patients	r = 0.71 p < 0.01	r = −0.02 *p* = 0.92	r = 0.95 *p* < 0.01
Colorectal patients	r = 0.87 p < 0.01	r = 0.37 *p* = 0.06	r = 0.86 p < 0.01
Heart Failure patients	r = 0.73 p < 0.01	r = 0.10 *p* = 0.61	r = 0.92 p < 0.01

### Ordinal composite outcome measure

The composite measure was significantly correlated with the standardized mortality rate (r = 0.78, *p* < 0.01) and the standardized long LOS rate (r = 0.98 p < 0.01) but not with the standardized readmission rate (Fig. 1). This is caused by the smaller variation in readmission rates, which is therefore weighted less in the variation of the composite. The strong correlation with long LOS may be caused by the larger number of events from this outcome than for the other outcomes which will therefore implicitly be weighted more – around 21% of patients overall were defined as having a long LOS, compared with 3% for mortality and 8% for readmission (Table 1). However, results were similar when using upper decile LOS (data not shown).

**Fig. 2:**
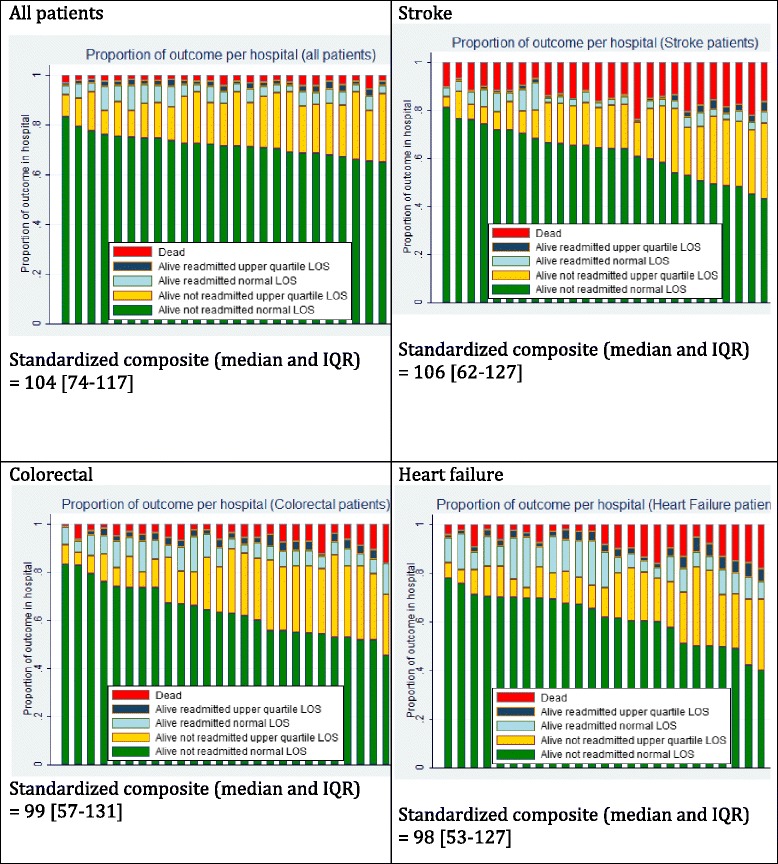
Crude outcome distribution per hospital, (*n* = 26) and standardized composite outcome (median and IQR)

Figure 2 shows the variation in the crude composite outcome between hospitals. This graphical presentation enables hospitals to see whether readmission rates are high after a normal or short LOS, which may indicate patients being discharged too early, or after a long LOS, which may indicate that these patients were sicker. The variation in the standardized composite outcome (median 104 IQR [74–117]) was in-between the variation in the individual outcome rates, being smaller than the variation in mortality rates (122 [72–132]) but larger than the variation in readmission rates (95[82–104]).

**Fig. 3:**
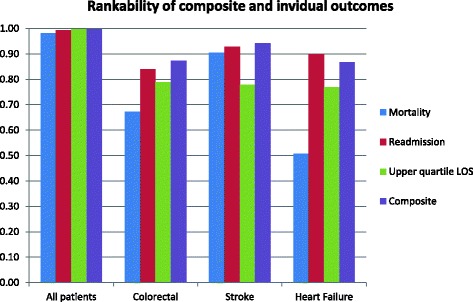
Rankability of composite versus individual outcomes

**Fig. 4:**
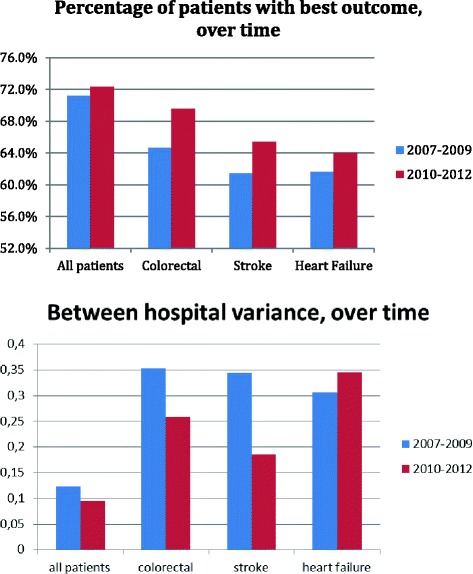
Changes in composite measure over time

Figure 3 shows that the reliability of ranking the hospitals (rankability) is similar or better for the ordinal composite measure than for individual outcomes. We used the composite measure in a post-hoc analysis to track changes over time to evaluate whether we have improved quality. As shown in Fig. 4, more patients were discharged alive in all 26 hospitals together, after normal LOS and without a readmission in the period 2010–2012 than in 2007–2009 (72.3% versus 71.2%, *p* < 0.001), particularly for colorectal patients (69.5% versus 64.6%, p < 0.001). This increase was due mostly to decreased mortality and shorter LOS. At the same time, the differences between hospitals (tau [2]) decreased, indicating more uniform outcomes across hospitals.

## Discussion

In this study, using a large international database, we have explored the interrelations between three common outcomes and derived a new ordinal composite measure. We found that hospital mortality and LOS rates were positively correlated at patient and hospital level. High readmission rates did not correlate with either mortality or long LOS rates. Our composite measure provides a rank-ordered view of the three outcomes and has comparable or better rankability than the individual outcomes, indicating that hospital comparisons with this composite measure are more reliable and stable.

Our results are consistent with the results from Krumholz et al. showing no association between hospital readmission and mortality rates [1]. Whereas they found a weak negative association for heart failure, the association in the present study was not significant among heart failure patients. Mortality and readmission rates may reflect different processes of care [6]. Early intervention and coordination of care in a hospital may be particularly important for mortality and length of stay, whereas the outpatient clinic and patient education may be factors influencing chances of readmission. At a patient level our results are consistent with previous studies with respect to an increased mortality and readmission risk in heart failure patients with longer length of stay [7] Contrary to previous studies, we examined both patient- and hospital level associations. These were particularly relevant for interpreting outcomes in stroke patients. It was shown that the hospitals with many patients dying were the same hospitals where the surviving patients had longer LOS. The same correlation was shown at both patient- and hospital-level for the other patient groups. So our results, together with the findings from Krumholz et al., suggest rather consistent patterns. Further, our results confirm that correlations between outcomes may differ between hospital and patient level [8], which argues against post-hoc combination different indicators at hospital level.

The proposed composite measure has substantive and statistical advantages. It combines multiple outcomes and therefore gives a more comprehensive view of quality of care. It incorporates the interrelations between the three outcomes, which prevents ‘gaming’ the single outcomes e.g. when hospitals receive incentives or penalties when individual outcomes are unfavorable. On the other hand, when hospitals use the composite measures to monitor their own performance, assessment of the constituent outcomes is always needed to identify areas for improvement, as with every composite measure. This adds to previous approaches to create composite measures e.g. combining outcomes with structure and process measures [9] [10], and that multiple outcomes are used, and that these are ordered [11].

The statistical advantage is that the composite outcome is ordinal and thus is more sensitive to between hospital differences than a dichotomous outcome. It contains more information; e.g. not only whether patients survived but also whether they were not readmitted. This explains why the rankability of the composite outcome was generally higher than of the single outcome. The rankability is a function of the differences between hospitals and the uncertainty in the estimates of the outcome per hospital. The latter is lower with the composite outcome, compared with single outcomes. The variation between hospitals might be smaller. In our example the variation in LOS was larger than in the composite measure, which explains that the rankability of LOS was somewhat higher. The variation in mortality was even larger. But as mortality is less frequent, the uncertainty in the estimates per hospital is large, and rankability is lower. Given a ‘true’ between-hospital variation, the rankability of composite ordinal outcome will always be higher compared with a dichotomous outcome.

The rankabilty in our study was high for the combined data but lower for some outcomes in the different diagnosis groups. Nevertheless rankability is substantially higher than in previous studies, showing the value of a large international database. Previous studies hardly ever reported rankability above 50% [3] [12] [13] [14] indicating that numbers of patients and events are typically just too low to reliably compare hospitals based on outcome. In the composite outcome, the most prevalent outcome, in this case LOS, will implicitly be weighted more. We performed sensitivity analysis with the upper decile LOS rather than the upper quartile LOS so that event rates were more equal to e.g. readmission rates. Similar results were found, although rankabilty was on average lower. Another possibility would be to define per diagnosis when LOS is considered too long and use that as a cut-off. Another possibility would be to put an explicit weight on the different outcomes. However, there is no evidence on how much more e.g. mortality should be weighted above readmissions after a long LOS, and in practice it will be difficult to determine explicit weights that appropriately represent the preferences of all different stakeholders.

The best category of this composite, an event-free hospital admission, obviously is what patients aim for. We also showed that within our collaboration as a whole, the percentage of patients with an event-free admission has increased over time particularly due to decreased mortality and shorter LOS. In addition, the variation between hospitals decreased over time. This is noteworthy as not all hospitals are faced with reimbursement penalties based on e.g. readmission rates, which may have caused variation to increase rather than to decrease over time. Part of the explanation may be the exchange of best practices within a collaboration among professionals, which occurred independently of these reimbursement policies.

Our study has some limitations, mostly due to the use of administrative data. Severely ill patients have higher chances of mortality, readmission and long LOS, and some hospitals may treat more of these severely ill patients than other hospitals. Our adjustment for severity of illness may have been insufficient, as suggested by others [15]. Part of the correlations between the outcomes that we observe might actually represent insufficient adjustment. With perfect adjustment the correlations are likely to be smaller. The same holds for using in-hospital mortality rates instead of 30-day mortality, which is known to affect hospital standardized mortality ratios [16]. If hospitals discharge patients early and they die outside of the hospital, this would result in shorter LOS, and lower mortality rates. Not counting these post-discharge deaths might thus have resulted in an overestimation of the relation between long LOS and mortality at a hospital level. We were only able to count readmissions to the same hospital, which will lead to underestimation of the readmission rate. Further, we did not explicitly study between-country differences while these may explain some of the between-center differences. But this was beyond the scope of the study.

## Conclusions

We created an ordinal composite measure to combine three commonly used outcome measures for quality of care: mortality, readmission and length of stay in a large international data set with information on patient characteristics and outcome. This composite measure gives a more comprehensive view of quality of care. In addition, the composite measure is more reliable and hence more useful to evaluate hospital performance.

## Abbreviations

CI: Confidence interval
GOAL: Global Outcomes Accelerated Learning
IQR: Interquartile range
LOS: Length of stay
OR: Odds ration
